# Urinary biomarkers of mycobacterial load and treatment response in pulmonary tuberculosis

**DOI:** 10.1172/jci.insight.136301

**Published:** 2020-09-17

**Authors:** Qianjing Xia, Myung Hee Lee, Kathleen F. Walsh, Kathrine McAulay, James M. Bean, Daniel W. Fitzgerald, Kathryn M. Dupnik, Warren D. Johnson, Jean W. Pape, Kyu Y. Rhee, Flonza Isa

**Affiliations:** 1 Weill Cornell Medical College,; 2 Center for Global Health,; 3 Department of Medicine, and; 4 Department of Microbiology & Immunology, Weill Cornell Medicine, New York, New York, USA.; 5 Memorial Sloan Kettering Cancer Center, New York, New York, USA.; 6 Les Centres GHESKIO, Port-au-Prince, Haiti.

**Keywords:** Infectious disease, Tuberculosis

## Abstract

**BACKGROUND:**

Control of the tuberculosis (TB) pandemic remains hindered in part by a lack of simple and accurate measures of treatment efficacy, as current gold standard markers rely on sputum-based assays that are slow and challenging to implement. However, previous work identified urinary *N^1^, N^12^*-diacetylspermine (DiAcSpm), neopterin, hydroxykynurenine, *N*-acetylhexosamine, ureidopropionic acid, sialic acid, and mass-to-charge ratio (*m/z*) 241.0903 as potential biomarkers of active pulmonary TB (ATB). Here, we evaluated their ability to serve as biomarkers of TB treatment response and mycobacterial load.

**METHODS:**

We analyzed urine samples prospectively collected from 2 cohorts with ATB. A total of 34 study participants from African countries treated with first-line TB therapy rifampin, isoniazid, pyrazinamide, and ethambutol (HRZE) were followed for 1 year, and 35 participants from Haiti treated with either HRZE or an experimental drug were followed for 14 days. Blinded samples were analyzed by untargeted HPLC-coupled high-resolution TOF-mass spectrometry.

**RESULTS:**

Urinary levels of all 7 molecules significantly decreased by week 26 of successful treatment (*P* = 0.01 to *P* < 0.0001) and positively correlated with sputum mycobacterial load (*P* < 0.0001). Urinary DiAcSpm levels decreased significantly in participants treated with HRZE as early as 14 days (*P* < 0.0001) but remained unchanged in cases of ineffective therapy (*P* = 0.14).

**CONCLUSION:**

Urinary DiAcSpm, neopterin, hydroxykynurenine, *N*-acetylhexosamine, ureidopropionic acid, sialic acid, and *m/z* 241.0903 reductions correlated with successful anti-TB treatment and sputum mycobacterial load. Urinary DiAcSpm levels exhibited reductions capable of differentiating treatment success from failure as early as 2 weeks after the initiation of chemotherapy, advocating its further development as a potentially simple, noninvasive biomarker for assessing treatment response and bacterial load.

**FUNDING:**

This work was supported by the Clinical and Translational Science Center at Weill Cornell College of Medicine (NIH/NCATS 1 UL1 TR002384-02 and KL2TR000458), the Department of Defense (PR170782), the National Institute of Allergy and Infectious Disease grants (NIAID T32AI007613-16, K24 AI098627, and K23 AI131913), the NIH Fogarty International Center grants (R24 TW007988 and TW010062), NIH grant (R01 GM135926), the Abby and Howard P. Milstein Program in Chemical Biology and Translational Medicine, and the Tuberculosis Research Units Networks (TBRU-N, AI111143).

## Abstract 2

A panel of biomarkers whose levels track with tuberculosis treatment efficacy and sputum mycobacterial load can be monitored non-invasively in urine.

## Graphical abstract

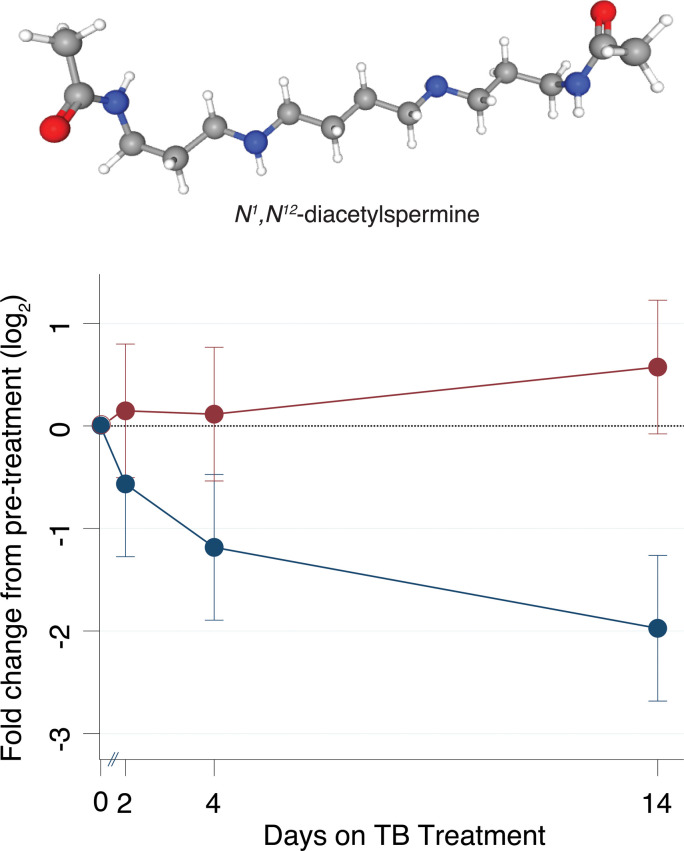

## Introduction

Tuberculosis (TB) remains the leading cause of death worldwide owing to a single infectious agent, and is the leading cause of death attributed to drug resistance (1). Control of the TB pandemic remains hindered in part by the limited range of clinically relevant diagnostic and treatment response biomarkers. Current gold standard methods for monitoring therapy response continue to depend on sputum-based assays (2). Such methods are recognized for their ability to indicate disease severity and transmissibility, but are limited by practical challenges associated with the ability to recover sputum, relative insensitivity of microscopy to bacterial burden and viability, and the inherently retrospective nature of culture-based methods, which often lag weeks to months behind the need for clinical decision making (3, 4). Recent advances in nucleic acid amplification-based methods such as GeneXpert have begun to overcome some of these barriers. However, despite their increased speed and sensitivity, such tests continue to require sputum samples, are unable to distinguish live from dead mycobacteria, and remain prohibitively expensive to operate in low- and middle-income countries, where more than 90% of TB cases occur (5, 6). These hurdles to the timely diagnosis of disease and verification of treatment efficacy are problematic because while awaiting test results, ineffective or only partially effective treatments continue to promote clinical progression, continued transmission, and the emergence of drug resistance itself (7, 8).

Sputum mycobacterial load is a widely recognized marker of disease severity that correlates with clinical symptoms, the presence of cavitary lung lesions, and transmission rates (9–11). Novel biomarkers of TB diagnosis and treatment response would ideally enable rapid detection and quantification of sputum bacterial load, and should be inexpensive, simple, noninvasive, and nonsputum based. Such characteristics would increase the ability to reach lower-resource health care systems and reduce cost of diagnostic algorithms. In addition to their potential to directly impact patient care, such biomarkers would also provide faster readouts of experimental drug efficacy that could accelerate TB drug development.

Urinary biomarkers have recently begun to emerge as clinically useful diagnostic markers of infectious disease and prognostic markers of treatment efficacy (12–14). Urine is an easily obtainable biological sample that is chemically complex, and indicative of host physiologic states, including infection (15). Moreover, growing evidence has demonstrated that TB may elicit specific patterns of immune activation, including unique transcriptional signatures and TB-specific T cell populations, some of which may be in the metabolic profiles of the blood and urine of afflicted patients (16–21).

In previous work, we identified *N^1^, N^12^*-diacetylspermine (DiAcSpm), hydroxykynurenine, neopterin, *N*-acetylhexosamine, ureidopropionic acid, sialic acid, and an uncharacterized molecule with mass-to-charge ratio (*m/z*) of 241.0903 as potential urinary biomarkers of active pulmonary TB (ATB) (Table 1) (22). These urinary molecule levels decreased after 60 days of anti-TB treatment in 20 participants, supporting their utility for indicating active TB disease (22).

Here, we investigated the potential of these same urinary metabolites to also serve as biomarkers of TB treatment response and mycobacterial load.

## Results

### Urinary molecule levels decrease during TB treatment.

**Figure 1:**
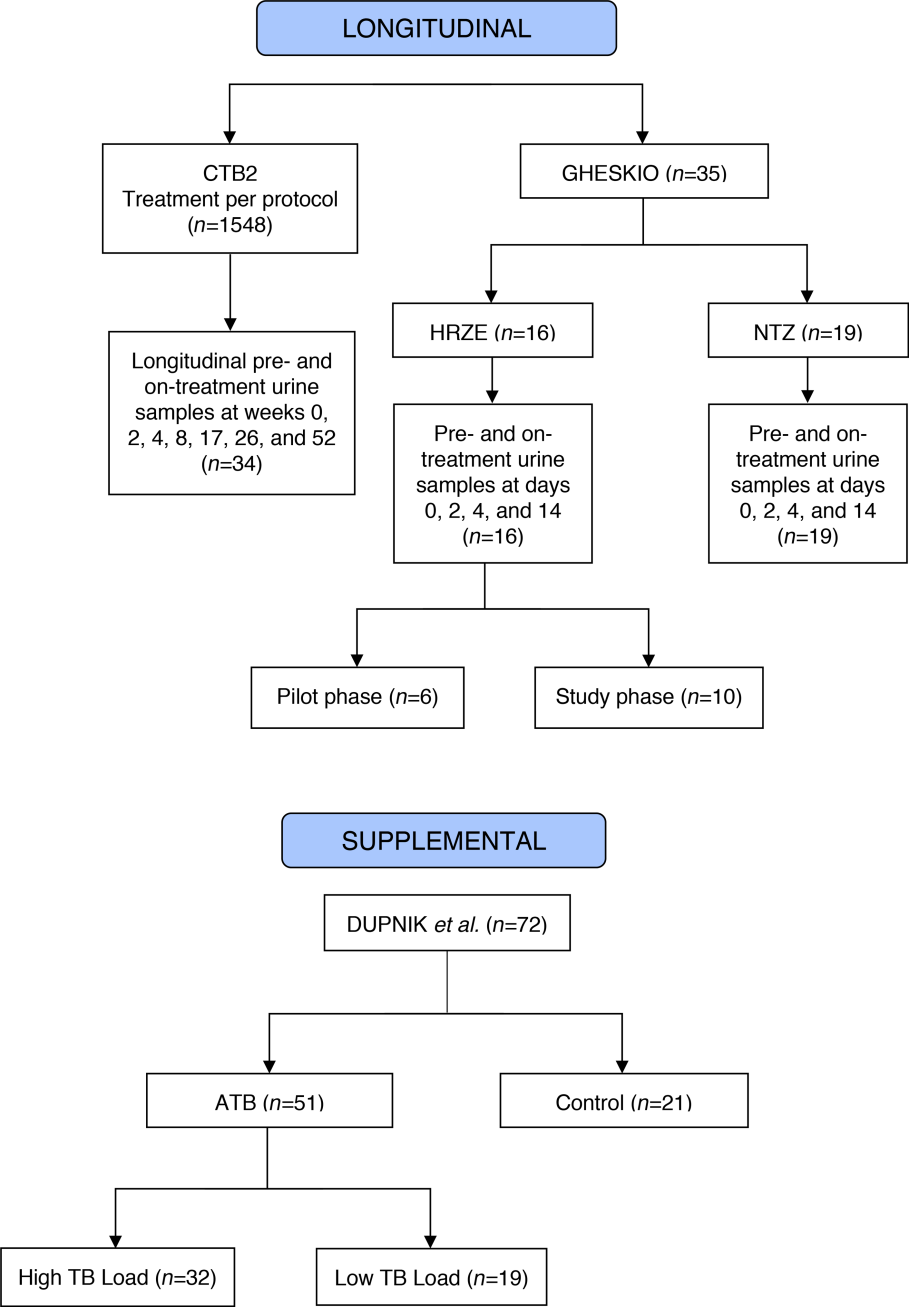
Study design flow chart of each study cohort. Two main longitudinal cohorts, CTB2 and GHESKIO, and 1 supplemental cohort were included in this study. CTB2, Consortium for TB biomarkers.

We first characterized the urine metabolic profiles of 34 participants treated for ATB using a blinded set of prospectively collected longitudinal urine samples from the REMoxTB trial (23) obtained through the Consortium for TB Biomarkers (CTB2) (Figure 1). All participants received either 8 weeks of isoniazid, rifampin, pyrazinamide, and ethambutol (HRZE), followed by 18 weeks of isoniazid and rifampin, or received moxifloxacin in combination with isoniazid or ethambutol as detailed in the study description (23). Table 2 shows available participant characteristics. All participants tested positive for TB by sputum culture, acid-fast bacilli (AFB) smear, or GeneXpert assay at the time of enrollment. All participants had negative AFB smears and sputum cultures at treatment termination (26 weeks) (Supplemental Table 1; supplemental material available online with this article; https://doi.org/10.1172/jci.insight.136301DS1). Participant urine samples were obtained before initiation of treatment (week 0) and at weeks 2, 4, 8, 17, 26, and 52 of the study. Samples were blinded, randomized, and normalized by dilution to 150 mOsm before untargeted metabolomic profiling by HPLC -coupled high-resolution TOF mass spectrometry (HPLC/MS).

**Figure 2:**
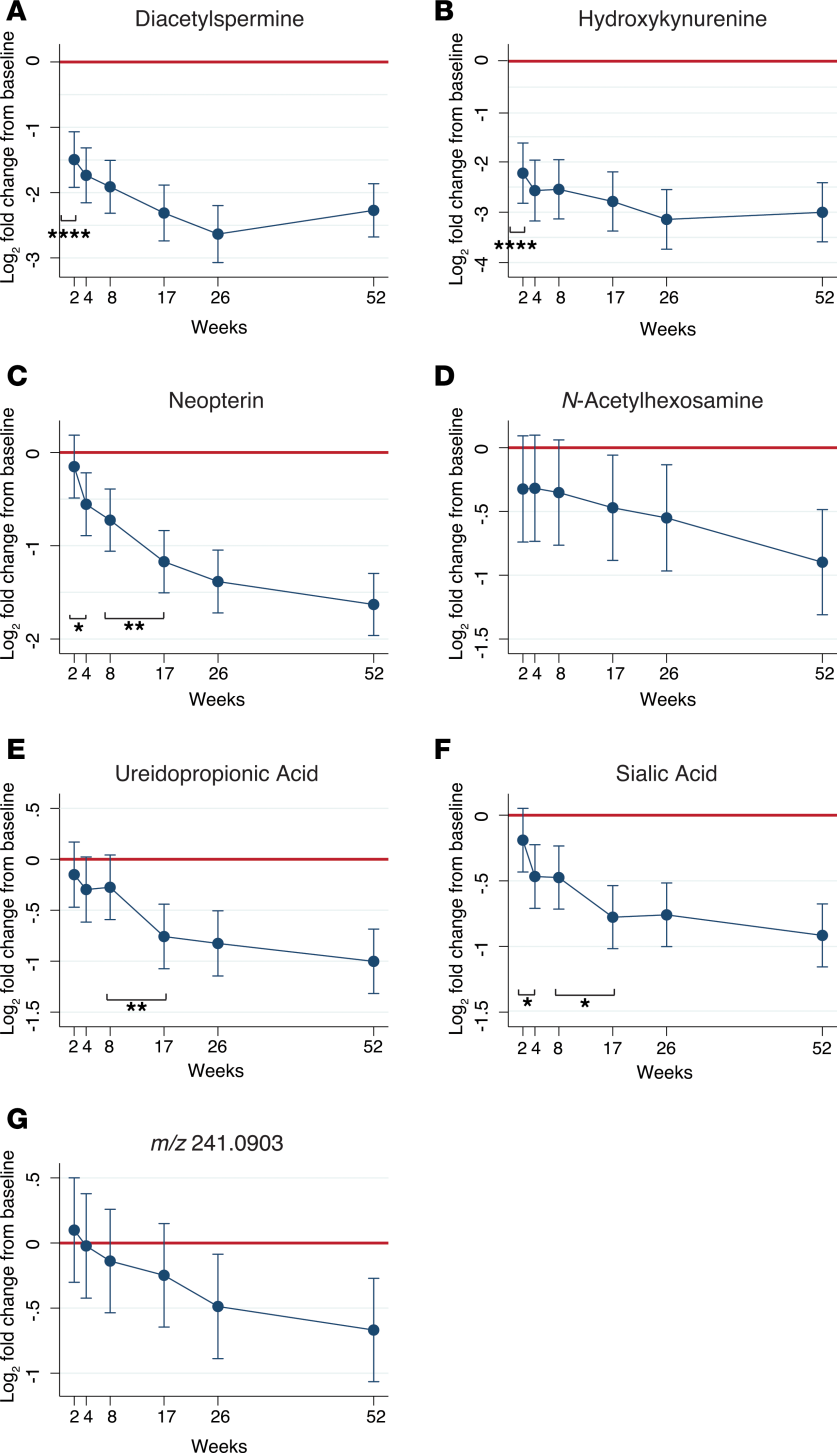
Molecule abundance decreased over course of treatment in clinically cured TB patients from CTB2 cohort (*n* = 34). (**A–G**) Mean fold change of each urinary molecule from baseline levels (week 0) are depicted in log_2_ scale. Red line represents no change from baseline. All original HPLC/MS molecule abundances were normalized to corresponding urinary creatinine levels. Error bars represent 95% CI. Statistical difference between adjacent time points was determined using the Wald test and represented by *. *P < 0.05, **P < 0.01, ****P < 0.0001. MS, mass spectrometry; TB, tuberculosis.

Urinary levels of DiAcSpm, hydroxykynurenine, neopterin, *N*-acetylhexosamine, ureidopropionic acid, sialic acid, and *m/z* 241.0903 all significantly decreased by the end of treatment at 26 weeks, after adjusting for age, sex, and BMI (linear mixed model, *P* = 0.01 to < 0.0001) (Figure 2). DiAcSpm and hydroxykynurenine levels significantly decreased after only 2 weeks of TB treatment (*P* < 0.0001, *P* < 0.0001, respectively) (Figure 2, A and B), whereas neopterin, *N*-acetylhexosamine, ureidopropionic acid, sialic acid, and *m/z* 241.0903 levels significantly decreased from baseline between 4 and 26 weeks of treatment (*P* = 0.01 to < 0.0001) (Figure 2, C–G).

### Urinary molecule abundance correlates with sputum mycobacterial loads.

**Figure 3:**
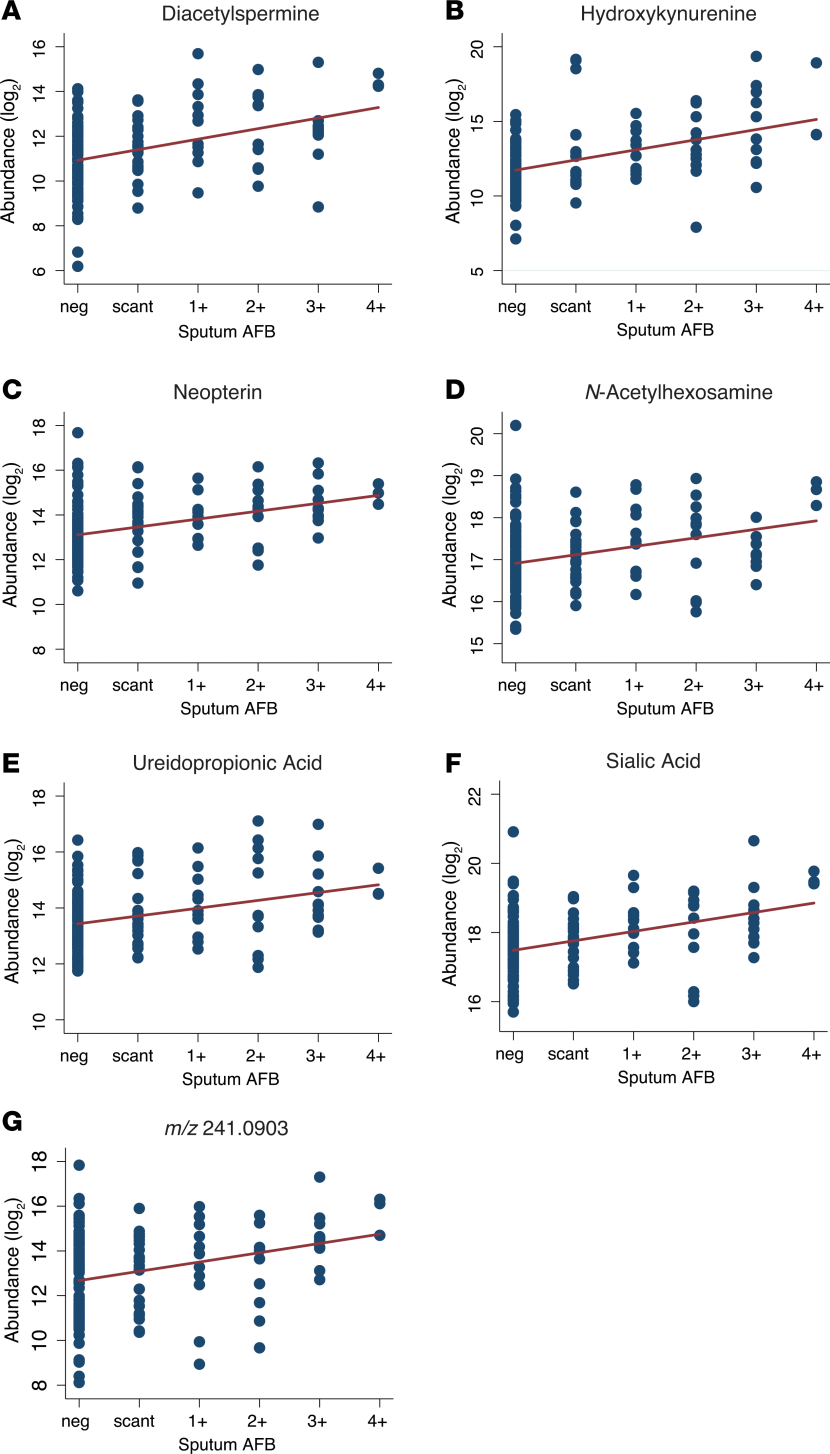
Urinary molecule abundance positively correlates with sputum mycobacterial burden in CTB2 cohort. (**A–G**) Scatterplots depict molecule abundances of each urine sample against its corresponding sputum acid-fast bacilli (AFB) score. Vertical axes represent HPLC/MS-measured molecular abundances in log_2_ scale after creatinine normalization. Data from all CTB2 participants (*n* = 34) and at all time points are represented. Regression lines adjusted for within-patient correlation and technical batch effects are represented in red (*P* < 0.0001, *r^2^* = 0.0928–0.2505).

We next investigated whether urinary levels of any of these 7 molecules correlated with sputum mycobacterial burden. Molecule abundance data from all CTB2 urinary samples with their corresponding sputum AFB scores are presented on scatter plots, regardless of participant identification or treatment time point, in Figure 3. Panel linear regression analysis was conducted for each of the 7 molecules accounting for intraparticipant correlation and batch effect of mass spectrometry (MS). Regression slopes identified strong positive correlations between sputum AFB score and molecule abundance for all 7 molecules (*P* < 0.0001).

**Figure 4:**
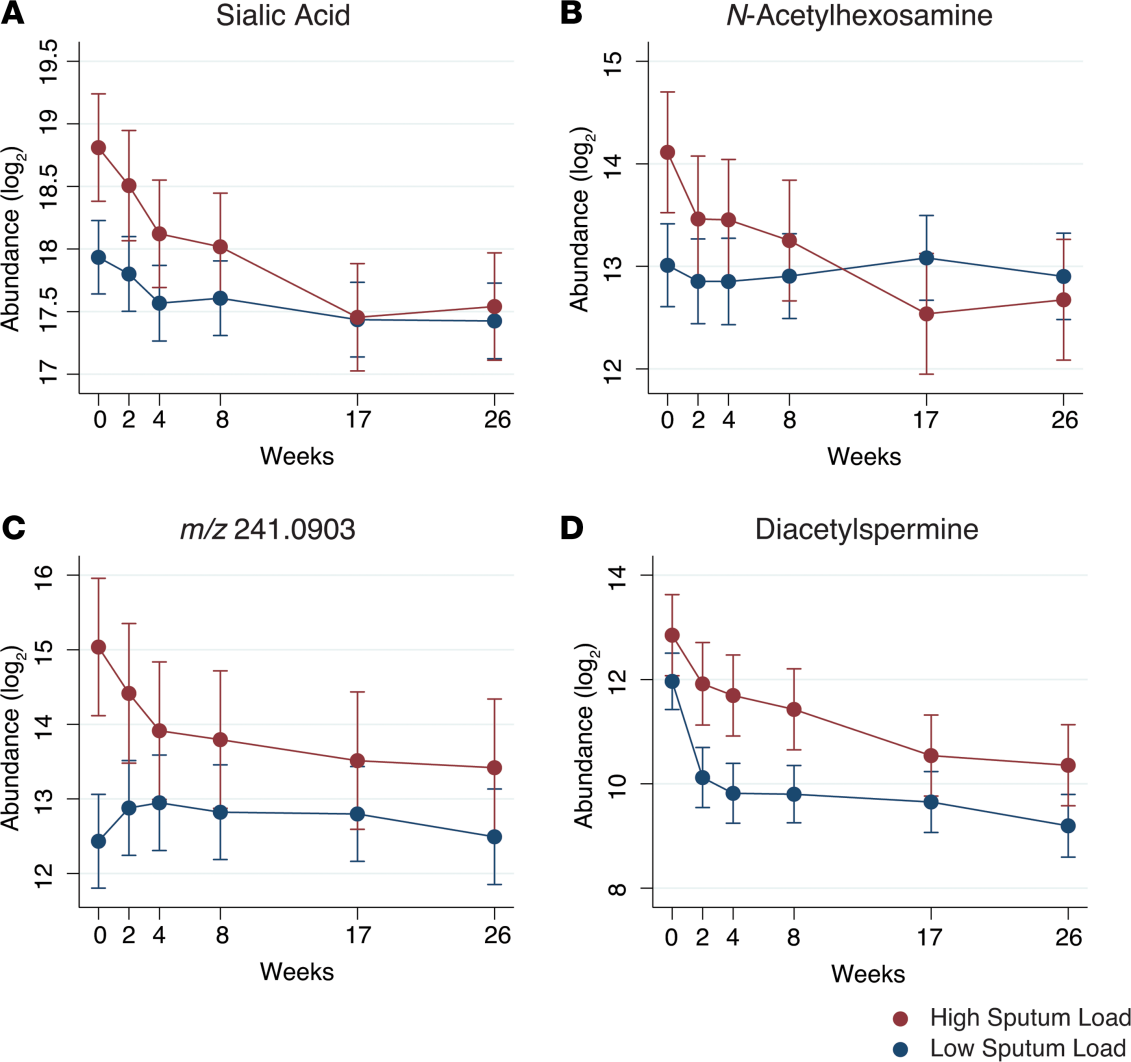
Mean urinary molecule abundance is higher in TB patients with high initial sputum mycobacterial load in CTB2 cohort. (**A–D**) Mean HPLC/MS abundance in log_2_ scale separated by initial sputum mycobacterial load of sialic acid (**A**), *N*-Acetylhexosamine (**B**), *m/z* 241.0903 (**C**), and diacetylspermine (**D**). Participants were separated by sputum AFB smear score at time of diagnosis (week 0). Initial AFB scores of 3+ or 4+ were categorized as high sputum load (*n* = 11, in red); initial AFB scores of 2+ or lower were categorized as “low sputum load” (*n* = 23, in blue). Error bars represent 95% CI. *m/z*, mass-to-charge ratio.

To further investigate the relationship between these molecule levels and treatment-induced declines in sputum bacterial load, we assigned each of the 34 CTB2 cases into “high” or “low” mycobacterial burden groups based on sputum AFB scores at the time of diagnosis (week 0). Participants with initial AFB scores of 3+ or 4+ were categorized as “high sputum load” (*n* = 11), and participants with initial AFB scores of 0, scanty, 1+, or 2+ were categorized as “low sputum load” (*n* = 23) (Supplemental Table 1). At baseline, levels of *N*-acetylhexosamine, sialic acid, and *m/z* 241.0903 differed significantly between high and low sputum groups (Figure 4, A–C). In all cases, higher initial mycobacterial burden was associated with higher urinary molecule abundance. In contrast, by the time of treatment termination at week 26, levels of all molecule from both “high” and “low” initial sputum load groups converged to similar levels, consistent with their shared clinical endpoint. DiAcSpm exhibited a similar trend to the 3 previously highlighted molecules, although the difference at baseline did not reach statistical significance (Figure 4D).

### DiAcSpm correlates with early treatment response outcomes and mycobacterial load.

To independently validate our findings, we obtained and analyzed urine samples from a second cohort of 35 participants with ATB enrolled in an early bactericidal activity (EBA) study at the Groupe Haitien d’Etudes du Sarcome de Kaposi et des Infections Opportunistes (GHESKIO) Centers in Port-au-Prince, Haiti. The study was designed to determine if the in vitro activity of the FDA-approved antiparasitic agent nitazoxanide (NTZ) against *Mycobacterium tuberculosis* (*Mtb*) could serve as a mycobactericidal agent for drug-sensitive pulmonary TB (24). Participants were recruited at the GHESKIO Centers and allocated into 2 treatment arms: 19 participants received NTZ for 14 days, whereas 16 participants received the standard HRZE TB treatment (Figure 1). Table 3 shows participant demographics. Inclusion criteria included either a sputum smear AFB score of at least 2+ or GeneXpert MTB/RIF positivity for MTB at the medium or high level at the time of enrollment. Urine samples were collected before treatment on day 0, and on days 2, 4, and, 14 after treatment initiation. All urine samples were blinded, randomized, and normalized to 150 mOsm before untargeted metabolomic analysis by HPLC/MS. Additionally, overnight sputum samples were collected from each patient upon diagnosis, and continuously collected every 2 days for 14 days to monitor for changes in culture time to positivity (TTP) as a measure of treatment efficacy (24). TTP data from this study were mathematically converted to CFU (25).

**Figure 5:**
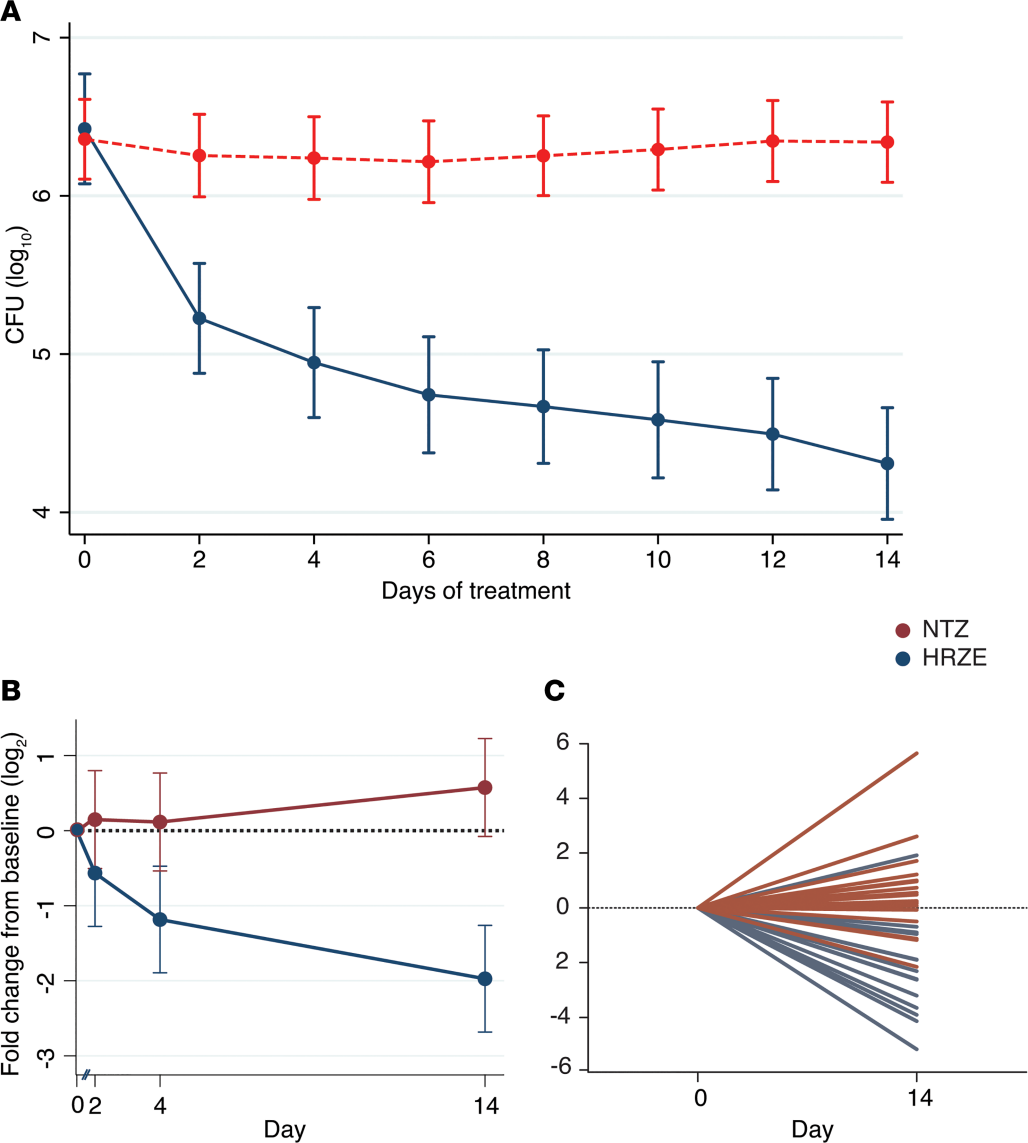
Urinary *N^1^, N^12^*-diacetylspermine (DiAcSpm) levels measured by mass spectrometry differentially decrease in successfully treated patients within the first 14 days in GHESKIO cohort. (**A**) Sputum culture CFUs show no change in bacterial burden of TB patients treated with 14 days of NTZ (*n* = 19, in red). CFUs decrease during treatment with rifampin, isoniazid, pyrazinamide, and ethambutol (HRZE) (*n* = 16, blue), demonstrating decreased bacterial burden. (**B**) HPLC/MS-measured urinary DiAcSpm decreases significantly in participants treated with HRZE (blue) but not in those treated with NTZ (red). Solid circles represent mean fold change from baseline levels in log_2_ scale. Error bars represent 95% CI and do not overlap at day 14. Dotted red line represents no change from baseline. (**C**) HPLC/MS-measured urinary DiAcSpm levels of individual participants. Each line represents an individual participant. Dotted line represents no change from baseline. All values have been normalized to corresponding urinary creatinine concentration.

Treatment with NTZ yielded no change in sputum culture CFU after 14 days, whereas treatment with HRZE resulted in the expected decrease in sputum culture CFU (Figure 5A). Therefore, these findings revealed NTZ lacks clinical antimycobacterial activity, making it possible to evaluate the performance of our urinary biomarkers in relation to treatment efficacy by comparing urine samples obtained both at the start and end of treatment from each arm of this study.

**Figure 6:**
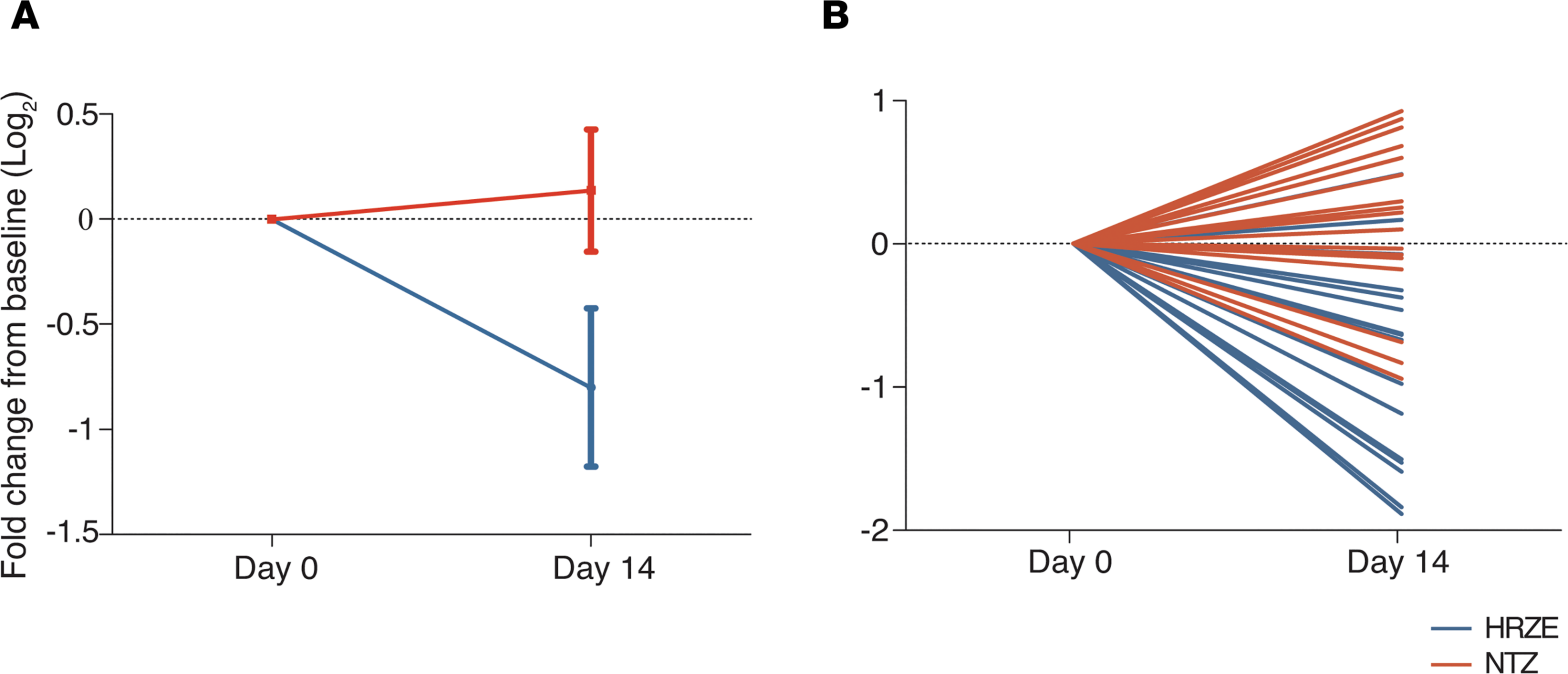
Change in urinary DiAcSpm levels in GHESKIO cohort confirmed using ELISA. (**A**) ELISA-measured urinary DiAcSpm levels demonstrate significant concentration decreases in participants treated with HRZE (*n* = 16, in blue) over 14 days, but not in those treated with NTZ (*n* = 19, in red). Solid dots represent mean fold change from baseline levels in log_2_ scale. Error bars represent 90% CI and do not overlap at day 14. (**B**) Changes in ELISA-measured urinary DiAcSpm levels of individual participants over 14 days. Each line represents a single participant. Dotted line in each graph represents no change from baseline. All values have been normalized to corresponding urinary creatinine concentration.

Linear mixed modeling of these data demonstrated that HPLC/MS-measured mean urinary DiAcSpm decreased significantly in the HRZE arm (*P* < 0.0001), but not in the NTZ arm (*P* = 0.14), and reached statistical significance at the study endpoint of treatment at day 14 (*P* < 0.0001) (Figure 5B). This trend was also observed on the individual patient level when comparing changes in urinary DiAcSpm between days 0 and 14 (Figure 5C). Moreover, these reductions could be detected using an analytically independent, commercially available monoclonal antibody-based ELISA (Trans Genic Inc.) developed for clinical use (26) (Figure 6, A and B).

Levels of urinary hydroxykynurenine, *N*-acetylhexosamine, ureidopropionic acid, and *m/z* 241.0903 showed similar significant decreases in mean abundance in participants treated with HRZE over the first 2 weeks, but did not achieve statistical significance when comparing the 2 treatment arms by the day-14 endpoint (Supplemental Figure 1). Linear mixed modeling of maximum daily axillary temperatures taken on corresponding treatment days (0, 2, 4, and 14) similarly failed to demonstrate significant difference between treatment arms (Supplemental Figure 2).

**Figure 7:**
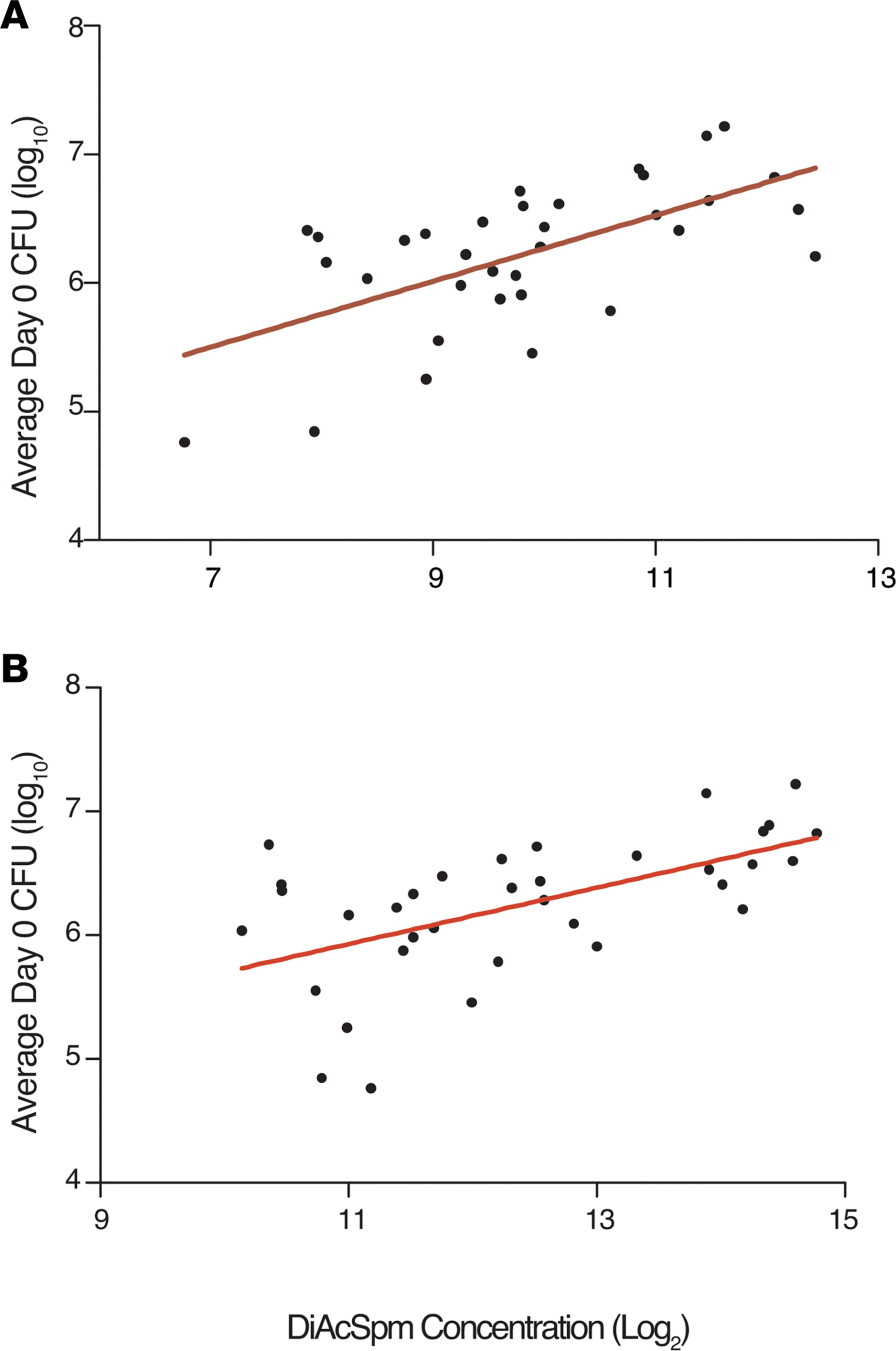
Urinary DiAcSpm concentration correlates with culture measures of mycobacterial burden in GHESKIO cohort. Scatterplots with regression lines show correlation between calculated CFU upon diagnosis (Day 0) and DiAcSpm concentration (*n* = 35). Increasing DiAcSpm concentration is associated with an increase in CFU, which is in turn a microbiological measure of mycobacterial burden. (**A** and **B**) DiAcSpm concentrations were determined using HPLC/MS chemical standard abundances (*P* = 0.0001, *r^2^* = 0.3812) (**A**) and ELISA (*P* = 0.0003, *r^2^* = 0.3318) (**B**). All values have been normalized to corresponding urinary creatinine concentrations.

Given the ability of DiAcSpm to differentiate effective HRZE from ineffective NTZ therapy, we further investigated the association between DiAcSpm and mycobacterial burden in this cohort. We plotted baseline (day 0) calculated culture CFUs against corresponding urinary DiAcSpm levels. As shown in Figure 7, DiAcSpm concentrations correlated positively with mycobacterial burden in both HPLC-MS and ELISA results, as indicated by higher corresponding CFU values (*P* = 0.0001 and 0.0003, *r^2^* = 0.3812 and 0.3318, respectively). This positive association was further validated using urine samples obtained from a third cohort, as previously reported by Dupnik et al. (16) (Figure 1 and Supplemental Figure 3).

**Figure 8:**
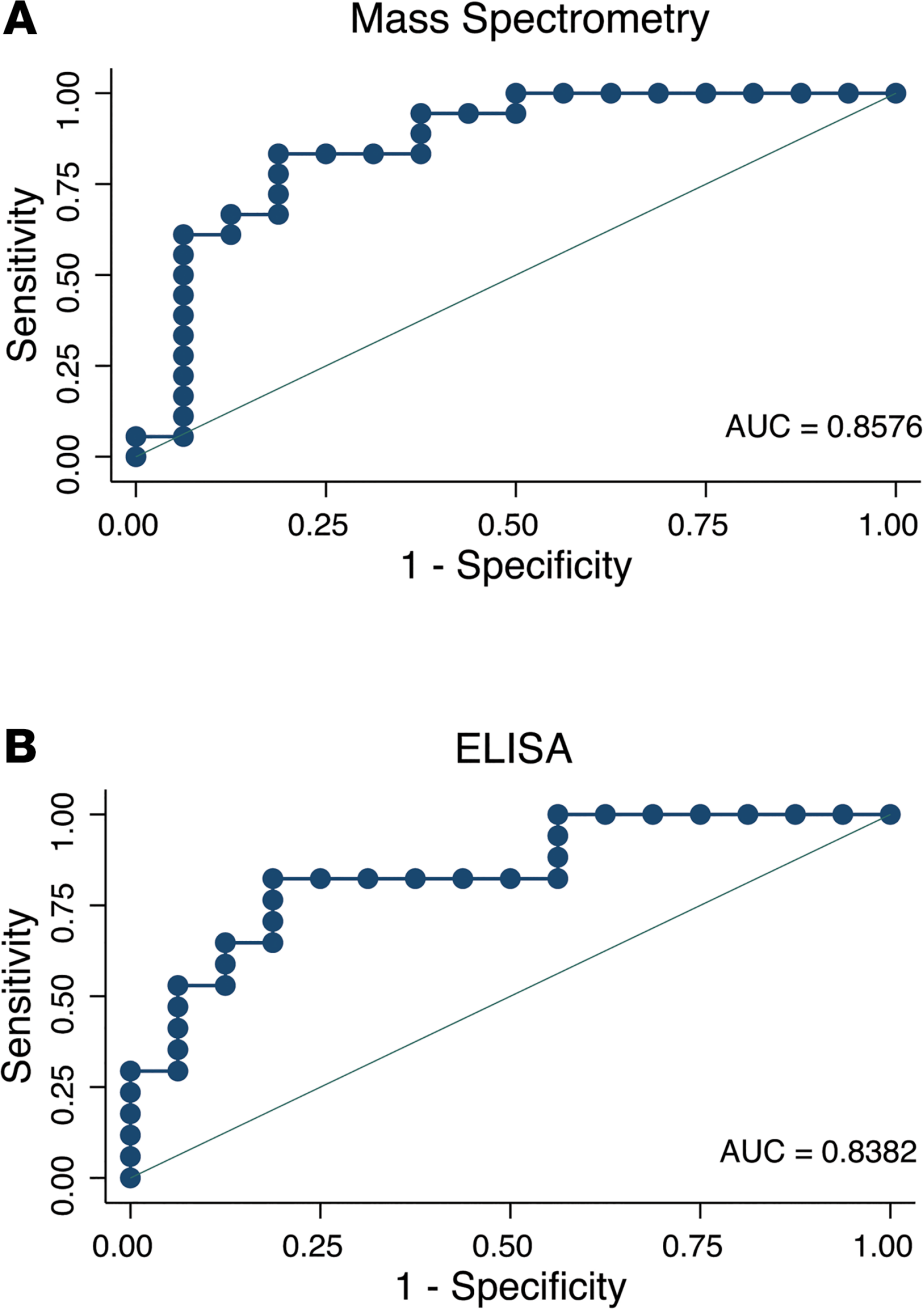
ROC curves show predictive value of urinary *N^1^, N^12^*-diacetylspermine using concentration fold change over 14 days. (**A** and **B**) ROC curves of participants in the GHESKIO cohort (*n* = 35) were plotted using urinary DiAcSpm concentration fold change between days 0 and 14 as classifiers to known participant treatment group (NTZ vs. HRZE). Area under the ROC curve (AUC) values are 85.76% (95% CI = 72.05, 99.48) and 83.82% (95% CI = 70.01, 97.64) for MS and ELISA-based readouts, respectively. AUC, area under the ROC curve; ROC, receiver operator characteristic.

To assess the predictive value of declining DiAcSpm concentration for treatment response, we constructed a receiver operator characteristic (ROC) curve using DiAcSpm concentration fold change at 14 days in NTZ- and HRZE-treated groups (Figure 8). Area under the ROC curve (AUC) values were 85.76% (95% CI = 72.05 and 99.48) and 83.82% (95% CI = 70.01 and 97.64) for MS - and ELISA-based data, respectively.

## Discussion

Current TB treatment response and disease burden measures remain rooted in sputum-based assays that are prohibitively slow, complex, and often incompatible with the health care settings in which TB is most frequently seen. Therefore, clinicians are often forced to rely on more subjective measures of symptom resolution while waiting several weeks or months for confirmation by sputum AFB and culture. Fast, sensitive, and affordable point-of-care tests in response to treatment thus constitute a major unmet clinical need that is critical for control of TB at both the individual and population levels (27).

### Urinary biomarkers decrease with treatment and correlate with mycobacterial load.

Biomarkers from human biofluids are useful reporters of host physiology in different pathological states; however, they are understudied in the context of TB treatment response. Our study demonstrates that urinary levels of DiAcSpm, hydroxykynurenine, neopterin, *N*-acetylhexosamine, ureidopropionic acid, sialic acid, and *m/z* 241.0903 all decreased over the 6-month course of treatment in 34 successfully treated ATB cases. Levels of these molecules predominantly stayed low or continued to decrease after treatment termination at 6 months. These findings, therefore, suggest that the molecular markers are not just measuring pharmacological actions of medication on host metabolism, but more importantly are reporting on disease activity itself. Previous work reported that urinary levels of these 7 molecules were elevated in ATB cases compared with cases of non-TB pulmonary disease with an overall sensitivity and specificity of more than 80% before initiation of treatment (22). Kynurenine, neopterin, and sialic acid levels have also been previously reported to be increased in various human ATB biofluids (28–33).

The rates at which each of these 7 urinary molecules decreased during 26 weeks of treatment varied. In all participants, some molecules dropped precipitously within the first 2 weeks, whereas others declined more gradually over the course of treatment. However, all 7 of these molecules were associated with significant reductions by the end of treatment, with some, such as hydroxykynurenine, exhibiting reductions as large as 8-fold (Figure 2B). These preliminary data suggest a potential role for 1 or a combination of these 7 urinary molecules to be developed into surrogate biomarkers of TB treatment response. Molecules exhibiting overall early declines could play a role in determining suitability of medical regimens, whereas molecules with overall slower kinetics may serve as longer term reporters of treatment efficacy. Binary classification of participants into high and low initial sputum burden groups also highlighted the differing rates of molecule decline based on initial disease severity (Figure 4). These findings raise the intriguing possibility of developing a number of clinically useful quantitative metrics of disease state and treatment response based on baseline levels of bacterial load, and absolute or relative reductions in biomarker levels.

Urinary levels of all 7 target molecules were also positively associated with sputum mycobacterial load. Levels of *N*-acetylhexosamine, sialic acid, and *m/z* 241.0903 were initially significantly higher in participants with high sputum AFB scores at diagnosis, but eventually converged with levels from participants with low sputum AFB by 26 weeks. Acetylated sugars such as *N*-acetylhexosamine are known components of the *Mtb* cell wall, and sialic acids are often expressed by pathogens to enhance intracellular survival and reduce host immune response (34). Therefore, it is possible that these molecules may reflect specific *Mtb*-derived products. Biological origin notwithstanding, changes in mycobacterial burden may be the best current indicator of treatment outcome for TB, and are routinely used in clinical practice to document treatment response (35). Correlative data between urinary molecule levels and corresponding sputum TB load presented in this study show promise for these urinary compounds to serve as surrogate markers of *Mtb* bacillary load.

### Potential role of DiAcSpm as a marker of antimycobacterial activity.

**Figure 9:**
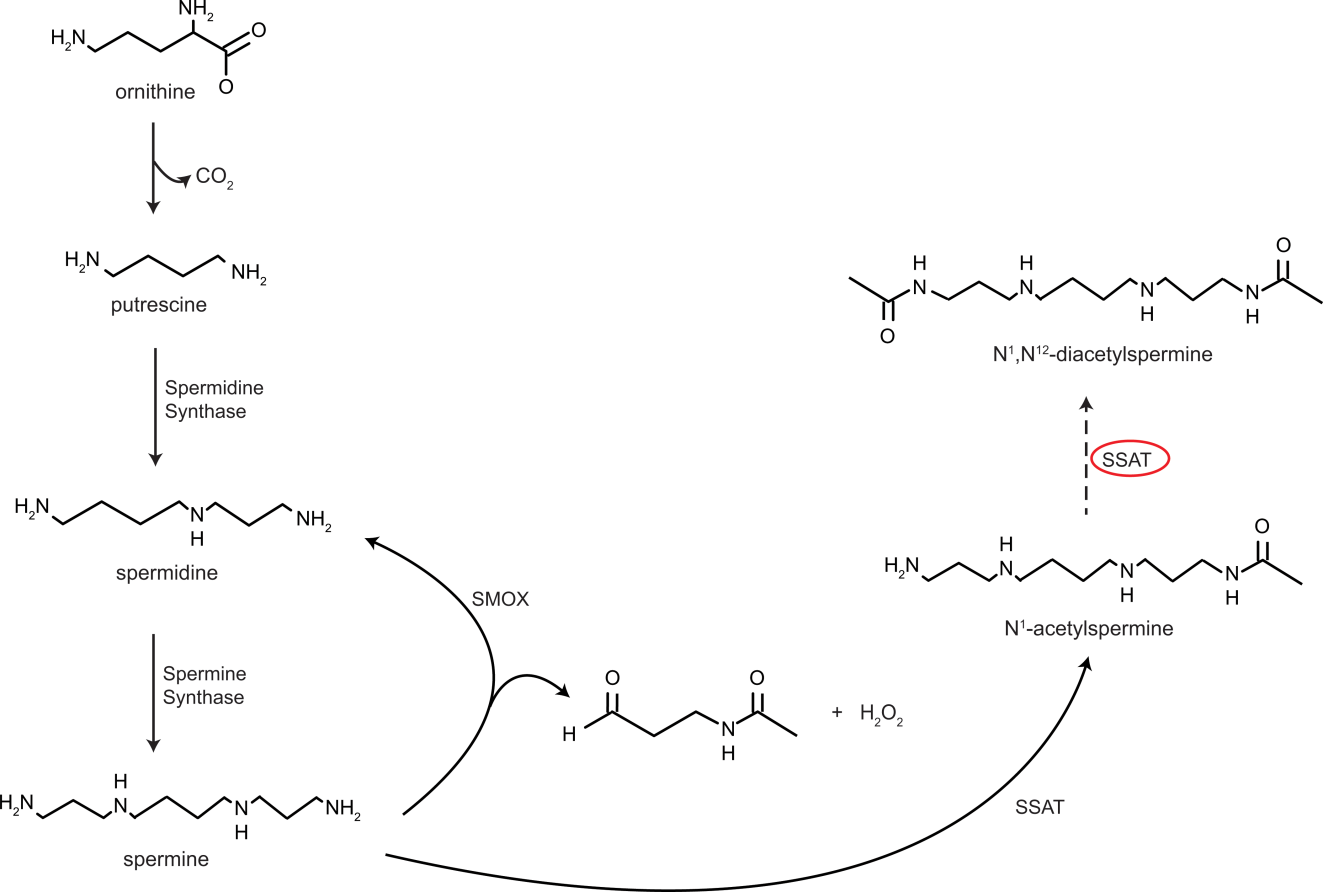
Polyamine synthetic and catabolic pathway. Circled enzyme spermidine/spermine *N^1^*-acetyltransferase (SSAT) is hypothesized to be responsible for the production of DiAcSpm through a second acetylation process. Solid lines represent known pathways; dotted line represents postulated pathway. SMOX, spermine oxidase.

Polyamines, including spermine, spermidine, and putrescine, are present in all organisms. They play important roles in major cellular processes such as growth and proliferation, and normally have tightly regulated intracellular levels (36). Current knowledge of polyamine metabolism is shown in Figure 9, which illustrates the ability of spermine to undergo catabolism either through direct oxidation by spermine oxidase (SMOX), or acetylation by spermidine/spermine *N^1^*-acetyltransferase (SSAT). *N^1^*-acetylspermine can undergo a second acetylation step via SSAT, forming DiAcSpm. Acetylated polyamines are then exported from the cell via an ATP-dependent polyamine transporter (36).

Considerable evidence has implicated polyamines in the pathogenesis of various mammalian bacterial diseases. In addition to DiAcSpm, several other metabolites in the polyamine catabolic pathway have previously been reported to be increased in TB states, including *N^1^*-acetylisoputreanine (37). Moreover, several bacteria have been shown to upregulate polyamine catabolism in infected host tissues, and it has been suggested that acetylated end products of polyamine catabolism facilitate cellular export (36, 38). Macrophages have conversely been implied as a source of DiAcSpm. A study by Hamaoki and Nagata revealed that peritoneal macrophages from lymphoid tumor-bearing mice produced DiAcSpm in the presence of exogenous spermine (39).Notably, an in vitro study from the 1950s demonstrated that exogenous spermine exhibited antimycobacterial properties after an unidentified enzymatic alteration (40). However, very little is known about the biological role of polyamines in TB immunopathogenesis (41, 42).

Previous work from our and other groups showed elevated levels of urinary DiAcSpm in ATB cases (22, 43). Additionally, our study demonstrates that DiAcSpm levels decrease rapidly with effective TB treatment. This could indicate that polyamine catabolism increases in TB-infected lung tissues and subsequently decreases with resolution of infection. An alternative explanation could involve an increased conversion of spermine to DiAcSpm by macrophages during active infection, in an attempt to produce antimycobacterial effects similar to those observed by Hirsch et al. in vitro (40). In this scenario, DiAcSpm levels would decrease with treatment considering that macrophages would face decreasing *Mtb* bacterial burden as the infection clears.

Our study also demonstrates the potential ability of DiAcSpm levels to differentiate treatment success from failure during the first 14 days of antimycobacterial therapy. The predictive value of a decrease in urinary DiAcSpm concentration was depicted using ROC curves, which showed AUCs of more than 80% in data obtained from 2 distinct detection modalities. The parameter used to construct ROC curves was the fold change in DiAcSpm concentration over time, which is independent of any specific concentration at any single time point. This finding highlights the predictive power of rate of change alone, regardless of starting molecule concentration, the latter of which has been shown to vary among individuals based on disease burden (Figure 3 and Figure 7). Interestingly, recent work has reported that clinical cure of TB may not be strictly equated with microbiologic sterilization (44–46), suggesting the presence of varying levels of residual bacteria among cured individuals. Therefore, the potential ability of DiAcSpm to serve as an early marker of treatment efficacy could significantly improve clinical medication management through its ability to detect potential treatment failures before the availability of drug susceptibility test results, and play an important role in facilitating EBA drug trials, which still rely on a time-consuming method of counting viable CFUs from sputum cultures.

From a translational perspective, DiAcSpm is a regular constituent of human urine, consistently accounting for 0.5% of total excreted urinary polyamines (47). DiAcSpm is not reabsorbed by the glomerular filtration system, and there is minimal diurnal variation in its urinary content among healthy individuals, suggesting tight control of its secretion (47, 48). These remarkable qualities, therefore, advocate further investigation of urinary DiAcSpm as a candidate biomarker of treatment response.

### Conclusion.

We have identified several candidate prognostic biomarkers of TB treatment response. Urinary DiAcSpm levels specifically show early and significant decrease in cases of successful TB treatment, suggesting its potential for development into an early biomarker of TB treatment efficacy.

## Methods

### Study design.

Longitudinal urine samples from 34 participants successfully treated for ATB were obtained from the CTB2 biorepository for urinary metabolite analysis over the course of treatment. Sputum mycobacterial data were made available to study correlation between urinary metabolites and *Mtb* burden. Additional urine samples were obtained from the GHESKIO Centers in Port-au-Prince, Haiti, from 35 participants enrolled in an EBA trial, and were used for urinary metabolite analysis in cases of ineffective treatment (24).

### CTB2 longitudinal cohort.

The CTB2, comprised of the Global Alliance for TB Drug Development, the TB Trials Consortium, and the AIDS Clinical Trials group, has created a collaborative biobank to accelerate biomarker discovery and validation for the diagnosis and treatment of TB. In collaboration with CTB2, we obtained prospectively collected longitudinal urine samples from 34 participants treated for confirmed ATB (Table 2). By request, clinical information for these samples were blinded to us until completion of metabolite analysis. Participants were recruited for 2 separate studies in unspecified African countries and followed for 1 year. Treatment consisted of either HRZE, followed by 18 weeks of isoniazid and rifampin, or was replaced in part by moxifloxacin as outlined in the REMox trial (23). Information on the specific treatment regimen corresponding to each participant, medication compliance, and drug susceptibility was not provided to us. Urine samples from each participant were collected at baseline (week 0), and at weeks 2, 4, 8, 17, 26, and 52 posttreatment. Sputum culture and AFB data were obtained at weeks 0, 4, 8, 26, and 52 posttreatment (Supplemental Table 1). Chest x-rays (CXRs) and GeneXpert (Cepheid) data were recorded at the time of diagnosis for 33 and 28 patients, respectively. All participants had either sputum AFB, culture, or GeneXpert positivity at time of diagnosis. All patients showed no culture or AFB positivity at treatment termination (26 weeks).

### Urine sample collection, storage, and shipment (CTB2).

Clean-catch urine samples were stored at –80°C in the Fischer BioServices facility in Bishop’s Stortford. Samples were shipped via PDP Couriers on dry ice, with constant temperature monitoring using a United Technologies Sensitech TempTale4 system, to the Belfer Research Labs at Weill Cornell Medicine and stored at –80°C until time of analysis.

### Assignment to sputum mycobacterial load group (CTB2).

AFB seen under smear microscopy are classified as 4+, 3+, 2+, 1+, scanty, or 0, with greater numbers denoting higher bacillary loads. To create a dichotomous variable for mycobacterial load, the 34 CTB2 cases were assigned into high or low mycobacterial burden groups based on sputum AFB scores at the time of diagnosis (week 0). Participants with initial AFB scores of 3+ or 4+ were categorized as high sputum load (*n* = 11), and participants with initial AFB scores of 2+, 1+, scanty, or 0 were categorized as low sputum load (*n* = 23).

### GHESKIO cohort.

Urine samples were collected from 35 participants with confirmed drug-sensitive ATB at the GHESKIO Centers as part of a 14-day EBA study of NTZ for the treatment of pulmonary TB (Table 3) (24). Urine was collected from each participant pretreatment on day 0, and on days 2, 4, and 14 of treatment. Participants were allocated into 2 treatment groups: 19 participants were treated with a 14-day course of NTZ, and 16 participants were treated with the standard HRZE therapy as defined by the WHO. Of the 16 HRZE-treated participants included in our analysis, 10 were randomized control participants from the clinical trial, and 6 were additional control participants enrolled in a pilot phase of the trial to validate laboratory assays. Overnight sputum samples from this cohort were collected every 2 days and cultured using the Mycobacterial Growth Indicator Tube automated liquid culture system (BACTEC; BD) to generate time-to-positivity () data (24). TTP data were subsequently mathematically converted to CFU values in this study, using the formula derived by Diacon et al. (25).

### Urine sample collection, storage, and shipment (GHESKIO).

Clean-catch urine samples were collected in sterile cups and immediately refrigerated at –4°C for 1–7 hours. Urine was then aliquoted on ice and stored at –80°C in GHESKIO facilities in Port-au-Prince, Haiti, until shipment to New York City. Shipments were sent on dry ice via World Courier from GHESKIO to the Weill Cornell Center for Global Health laboratory in New York City, and stored at –80°C until time of analysis.

### Sample preparation.

Samples from all cohorts were stored in a –80°C freezer at the Belfer Research Building at Weill Cornell Medicine until testing. Samples were blinded, randomized, and prepared in sets of 20–25. The osmolality of each sample was measured using an Advanced Instruments model 3250 Micro-Osmometer. Samples were then centrifuged for 10 minutes at 10,000 rpm in PALL nanosept centrifuge devices. Filtered substrate was diluted with MilliQ water to 150 mOsm to standardize the salt concentration within each sample. All samples below 150 mOsm before dilution were excluded from analysis. Diluted samples were mixed with liquid chromatography-MS (LC/MS) grade methanol containing 0.2% formic acid at a 1:1 sample to solvent ratio for HP analysis. Each set of 20–25 samples was run with a standard solution that consisted of 10 μM glutamate, succinate, lysine, and nicotinic acid that served as analytical quality control standards for the LC/MS. Pooled urine samples were included periodically throughout each set to allow for normalization of peak intensities and monitoring of mass spectrometer sensitivity within each run. One-third of the total urine samples from both CTB2 and GHESKIO cohorts were randomly selected for replicate runs to ensure data reproducibility. Replicate runs were performed using previously unthawed urine aliquots, independently randomized, and run in sets of 20–25 samples with the previously described standard solutions.

### HPLC/MS analysis.

Samples were analyzed using an Agilent Technologies 6230 TOF LC/MS. LC separation was achieved using a Cogent 4 Diamond Hydride column with an initial gradient of 85% LC/MS grade acetonitrile containing 0.2% formic acid, followed by a gradual increase in hydrophilicity to 95% LC/MS grade water containing 0.2% formic acid. Detected ions were indexed and characterized using their ion *m/z* and chromatographic retention time. Data were analyzed using Agilent Technologies Qualitative Analysis B.07, Agilent Technologies MassHunter Profinder B.08, and XCMS software. Compound identification was achieved using known *m/z* and retention time coupled to chemical standards of targeted compounds run with each set of urine samples. Identity of DiAcSpm was further confirmed using MS/MS fragmentation analyses of chemical standards and random patient urine samples. DiAcSpm chemical standards at 5 known concentrations (50 nM, 100 nM, 500 nM, 1 μM, and 5 μM) were included within each run to create standard curves for urinary DiAcSpm concentration calculation.

### Urinary DiAcSpm ELISA kits.

Previously unthawed urinary samples were used for this portion of the analysis. A total of 50 μL vortexed urine was centrifuged for 5 minutes at 1500 rpm. Urine was serially diluted 4 to 9 times for resulting concentrations to remain within ELISA kit detection range. Absorbance at 490 nm was measured using a Spectramax M2 microplate reader. Each sample was measured in duplicates, and all measured DiAcSpm concentrations were within range of the standard curve. Final results were adjusted for initial dilution ratio and further normalized to respective urinary creatinine concentrations, with a final unit of nmol/g creatinine.

### Creatinine normalization.

All molecule abundances were additionally normalized to creatinine concentrations of corresponding urine samples using a creatinine colorimetric assay kit (MilliporeSigma, catalog MAK080). Absorbance at 570 nm was measured using a Spectramax M2 microplate reader. Each sample was measured in duplicates, and all measured creatinine concentrations were within range of the standard curve.

### Longitudinal analysis.

Longitudinal trends of target urinary molecules were fitted using a mixed model. We estimated the effects of treatment at each time point as fixed effects while incorporating subject-specific abundances as random effects in the model. Hypotheses of factor variables and their interactions were assessed using the Wald test provided by the STATA margins command.

### CTB2 cohort.

We used a binary variable (high vs. low initial sputum AFB) to estimate the effect AFB had on longitudinal abundance profiles in this cohort. We included interaction terms between AFB and time to account for confounding trends over time. Time and AFB effects were calculated while adjusting for BMI, age, and sex.

### GHESKIO cohort.

We used the 2 treatment arms of NTZ and HRZE as binary variables in our statistical model. Interactions between treatment and time were included and assessed. Treatment and time effects were calculated while adjusting for age and baseline weight.

### Molecule abundance correlation with sputum AFB Score (CTB2).

We conducted panel linear regression analyses using per patient trend profiles of sputum AFB as covariates (Supplemental Figure 4), and corresponding per patient trend profiles of each biomarker as outcomes (Supplemental Figures 5 and 6). This per patient aggregate analysis of the correlation between AFB and urinary molecule levels allowed us to account for within patient correlations and mass spectrometer technical batch effects. Data from all available CTB2 cohort samples and time points were included.

### GHESKIO cohort TTP and CFU.

Effect of treatment (NTZ vs. HRZE) was assessed by fitting a mixed model on longitudinal arrays of CFU data. CFU values were mathematically converted from clinically measured patient TTP data, using the formula log_10_(CFU) = 16.41 – 5.17 * log_10_(TTP) derived by Diacon et al. (25). Graphical representation of the original TTP data was previously shown in a study by Walsh et al. (24).

### Dupnik cohort urine DiAcSpm correlation with GeneXpert sputum load.

We obtained banked urine samples collected from participants originally described by Dupnik et al. in a study reporting on blood transcriptomic markers of sputum mycobacterial load (16). This cohort consisted of 51 individuals with active pulmonary TB and 21 community controls with no signs or symptoms of TB and no prior history of TB. Of the 51 participants diagnosed with TB, 19 had low TB load and 32 had high TB load as determined by GeneXpert MTB/RIF C_T_ values. Further details on participant selection, sample collection, and experimental group assignments are further described in the Methods section of Dupnik et al. (16). Urine samples obtained as part of this study were stored at –80^o^C in the Belfer Research Labs at Weill Cornell Medicine until time of analysis. Sample preparation and analysis on the HPLC/MS were completed as described in earlier sections. We used a 1-way ANOVA test to determine whether a statistically significant difference existed between DiAcSpm concentrations of participants within the control, low sputum, and high sputum groups. We further used 2-tailed Welch-corrected *t* tests to determine differences between each 2 adjacent categories (i.e., control vs. low sputum group and low vs. high sputum group).

### Statistics.

All normalized molecule abundances were log_2_ transformed for analysis and visualization. Data analysis was performed using STATA SE version 15 and GraphPad Prism 6. For all statistical analyses, a *P* value of less than 0.05 was considered significant by 2-tailed Student’s *t* tests.

### Study approval.

Consent was obtained from all participants by local health workers during meetings conducted in their local language. All participants provided written informed consent before inclusion in the clinical cohort studies. IRB approval was obtained for the present study at Weill Cornell Medicine, New York, New York, USA. Studies from which CTB2 and GHESKIO samples were obtained have IRB approval at their respective institutions.

## Author contributions

FI and QX designed and conducted the experiments, acquired and interpreted MS data, and evaluated urinary molecule performance as markers of successful TB treatment. MHL performed statistical analyses. JMB, KFW, KM, and KMD collected and provided clinical samples and clinical data. KYR provided reagents and machinery for the study. QX, FI, and KYR wrote the manuscript. KYR and DF supervised and coordinated the work. WDJ, JWP, and DWF contributed to the clinical study design and samples from the GHESKIO center. All authors reviewed the manuscript, agreed with the results, and provided insight.

## Supplementary Material

Supplemental data

ICMJE disclosure forms

## Figures and Tables

**Table 1:**
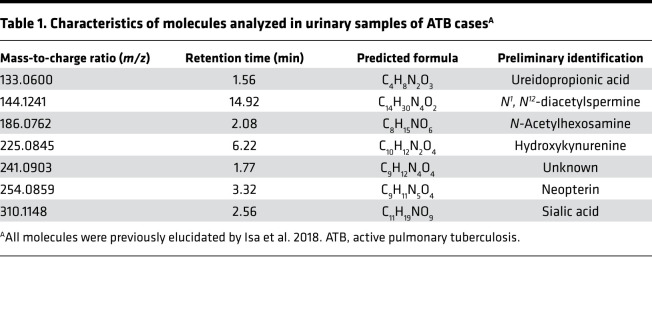
Characteristics of molecules analyzed in urinary samples of ATB cases^A^

**Table 2:**
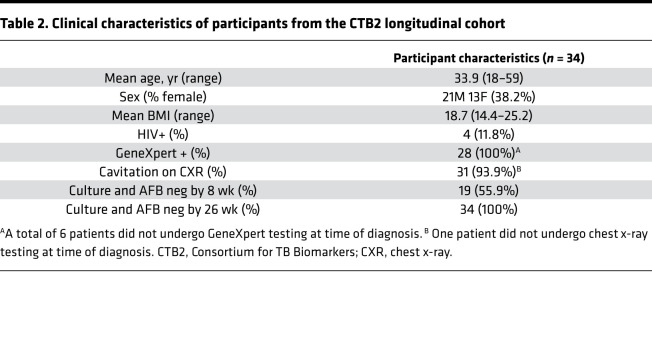
Clinical characteristics of participants from the CTB2 longitudinal cohort

**Table 3:**
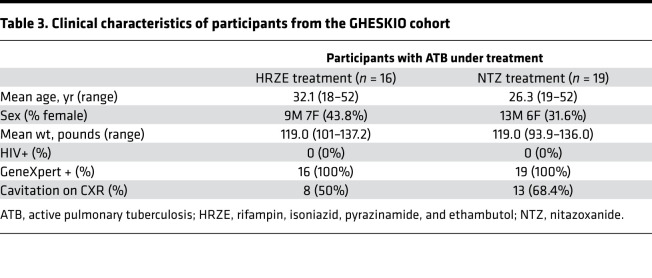
Clinical characteristics of participants from the GHESKIO cohort

## Version 1 08/18/2020

In-Press Preview

## Version 2 09/17/2020

Electronic publication
