# Supplementary material for: Urinary biomarkers of mycobacterial load and treatment response in pulmonary tuberculosis
Source: JCI Insight. 2020 Sep 17;5(18):e136301. doi: 10.1172/jci.insight.136301 (PMC7526545; doi:10.1172/jci.insight.136301)
Supplement: Supplemental data [file jciinsight-5-136301-s147.pdf]

Supplementary Information

Urinary Biomarkers of Mycobacterial Load and Treatment Response in Pulmonary Tuberculosis

Qianjing Xia MD, Myung Hee Lee PhD, Kathleen F. Walsh MD, Kathrine McAulay PhD, James M. Bean PhD, Daniel W. Fitzgerald MD, Kathryn M. Dupnik MD, Warren D. Johnson MD, Jean W. Pape MD, Kyu Y. Rhee MD PhD, and Flonza Isa MD

Figure S1 ……………………………………………………………………….Page S2

Figure S2 ……………………………………………………………………….Page S3

Figure S3 ……………………………………………………………………….Page S4

Figure S4 ……………………………………………………………………….Page S5

Figure S5 ……………………………………………………………………….Page S6

Figure S6 ……………………………………………………………………….Page S7

Table S1 …………………………………………………………………….….Page S8

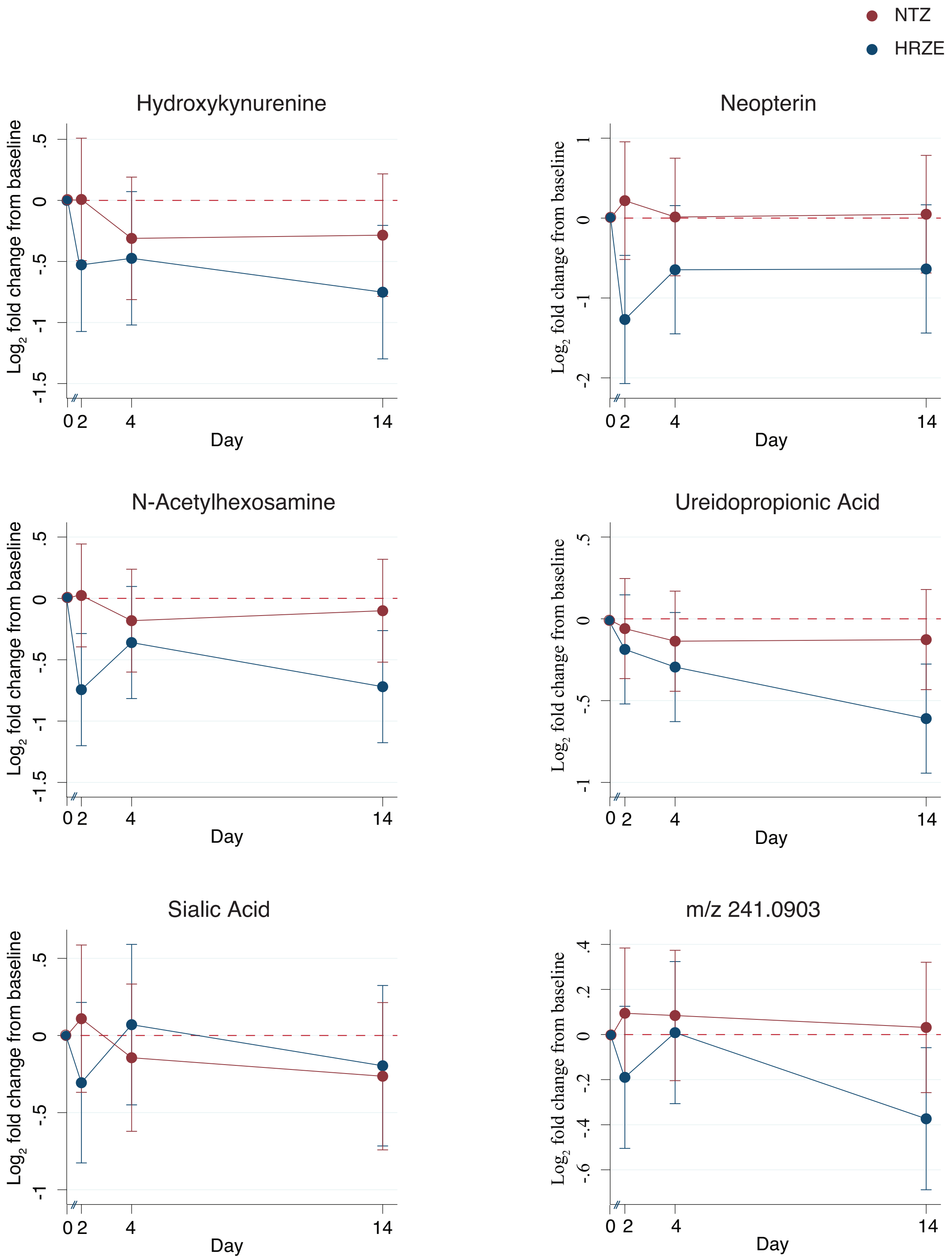

Figure S1. Mean log₂ fold changes of remaining six urinary molecules in participants treated with NTZ (n=19, red) and HRZE (n=16, blue) in GHESKIO cohort (n=35). HPLC/MS-measured urinary levels of target molecules depicted uring mean fold change from baseline levels (Day 0). Error bars represent 95% CI. Horizontal dashed line represents no change from baseline.

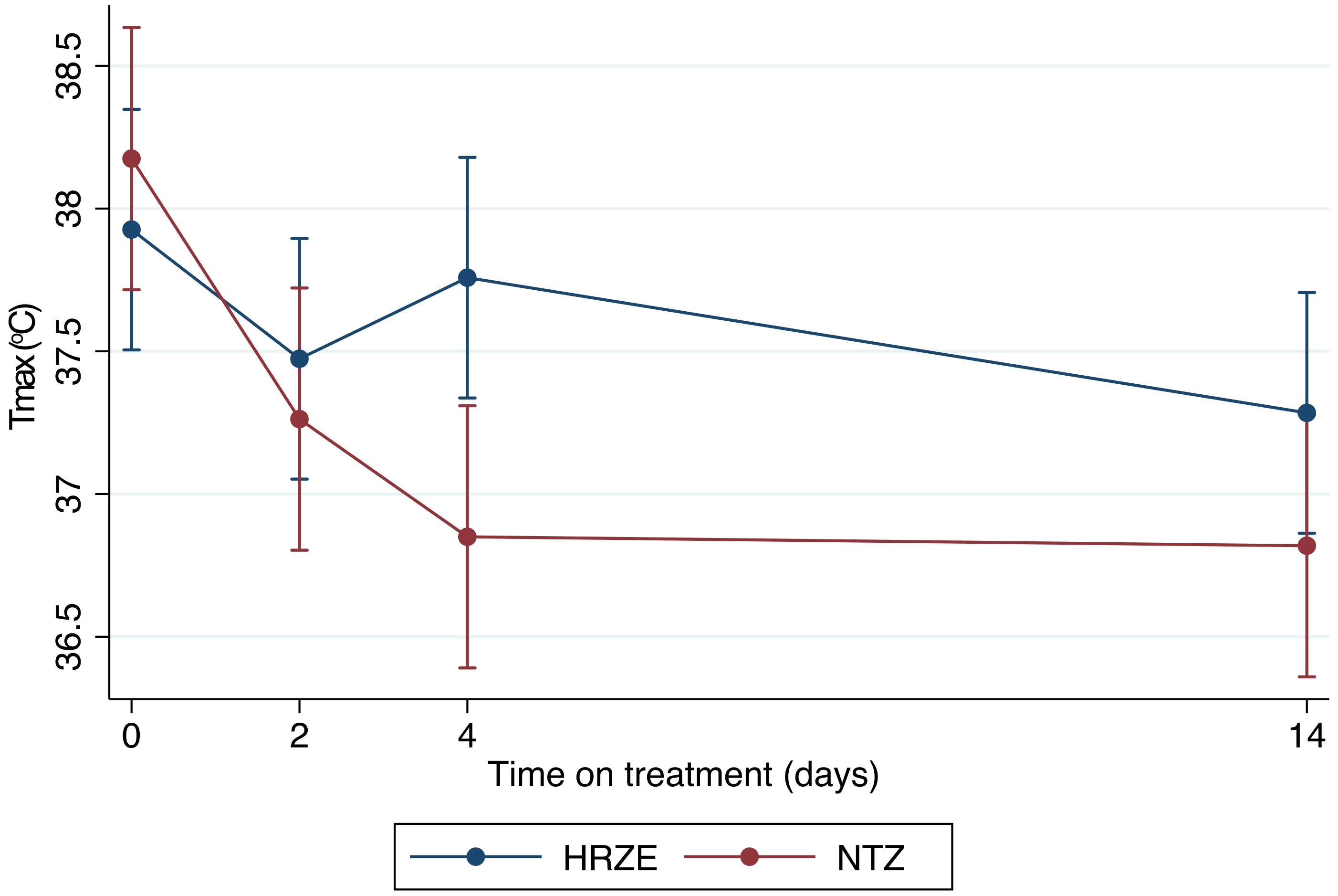

Figure S2. Longitudinal body temperature measurements do not significantly differ between TB treatment success and failure groups in GHESKIO cohort (n=35). Maximum daily axillary temperatures showed an overall decreasing trend regardless of treatment group. Time points used in this figure correspond to those of urine collection (days 0, 2, 4, and 14). Error bars represent 95% CI.

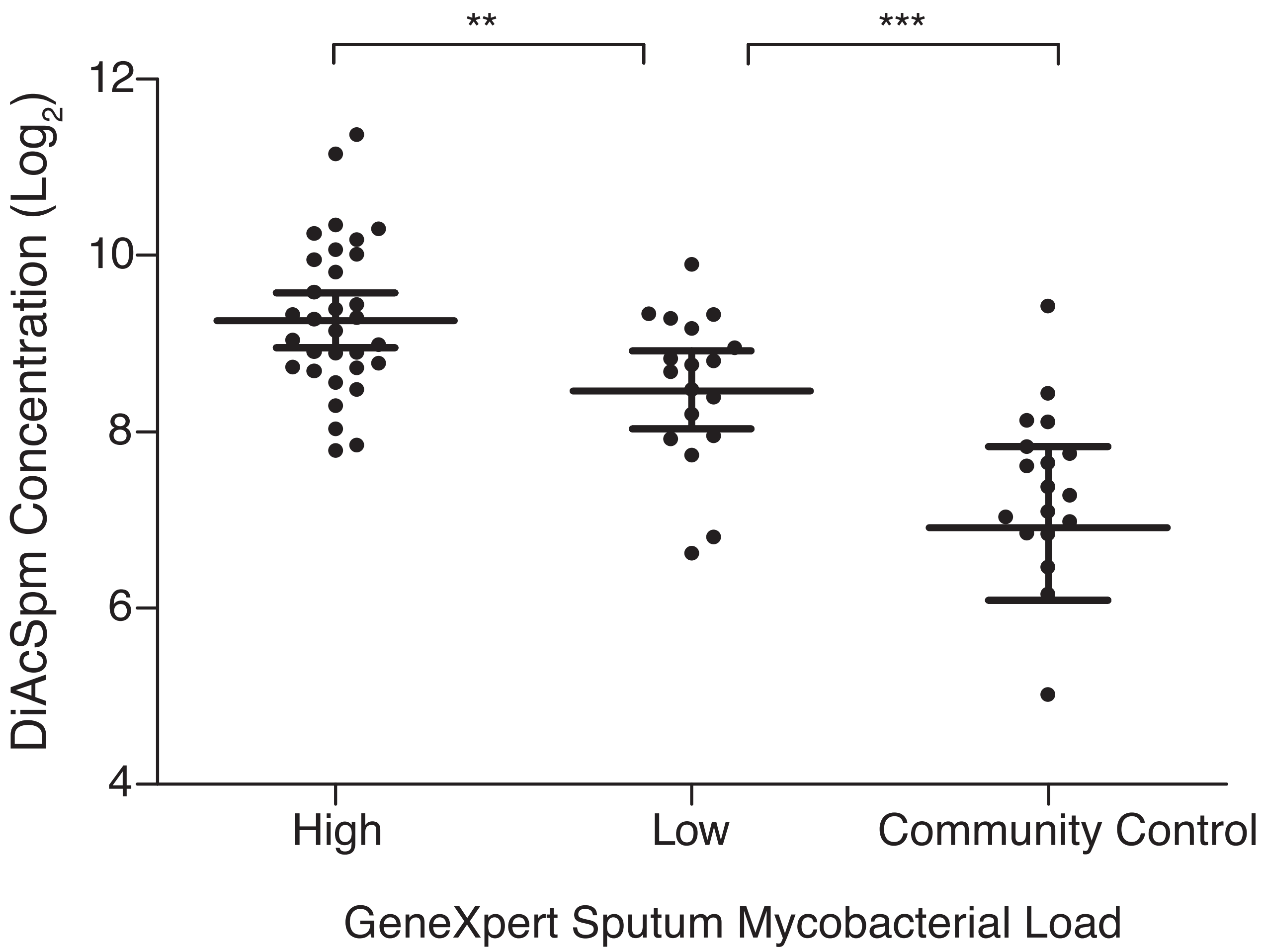

Figure S3. DiAcSpm levels associate with sputum mycobacterial load in third cohort. In this cohort of 51 ATB cases divided into high (n=32) and low (n=19) sputum mycobacterial loads, DiAcSpm significantly differed between the two sputum load groups (p=0.0038), and between low sputum load and healthy community controls (n=21) (p=0.0008). Dichotomous variable for mycobacterial load was determined using GeneXpert scores as defined by Dupnik et al. (16). DiAcSpm concentrations were determined using HPLC/MS chemical standard abundances. All values have been normalized to corresponding urinary creatinine concentrations. One-way ANOVA $p<0.0001$. Statistical difference between adjacent categories was determined using two-tailed Welch-corrected t-test with significance represented by *. (**, $p<0.01$; ***, $p<0.001$)

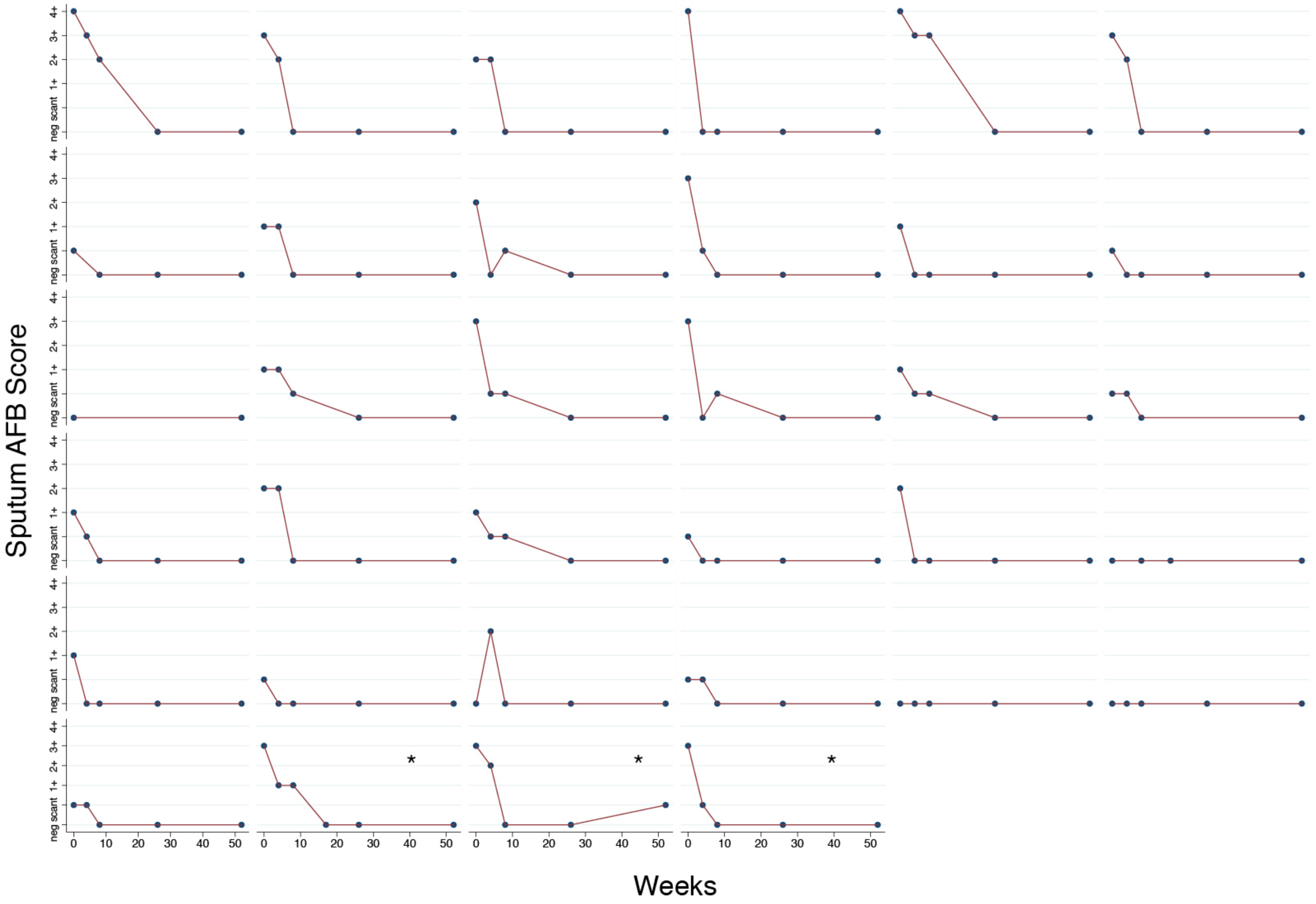

Figure S4. AFB Score Trend over time by Patient in CTB2 Cohort (n=34). Graphs of individual patients show sputum AFB score trend profiles over time rom the CTB2 cohort. Trends are generated using available AFB score data. The last three graphs marked with "*" represents graphs of patients who showed repeat positive culture or AFB scores at week 52 after treatment termination (Table S1). Lack of clinical data informing whether the positivity at 52 weeks was due to re-infection, incomplete treatment, or other causes prevents meaningful interpretation of these findings at this time.

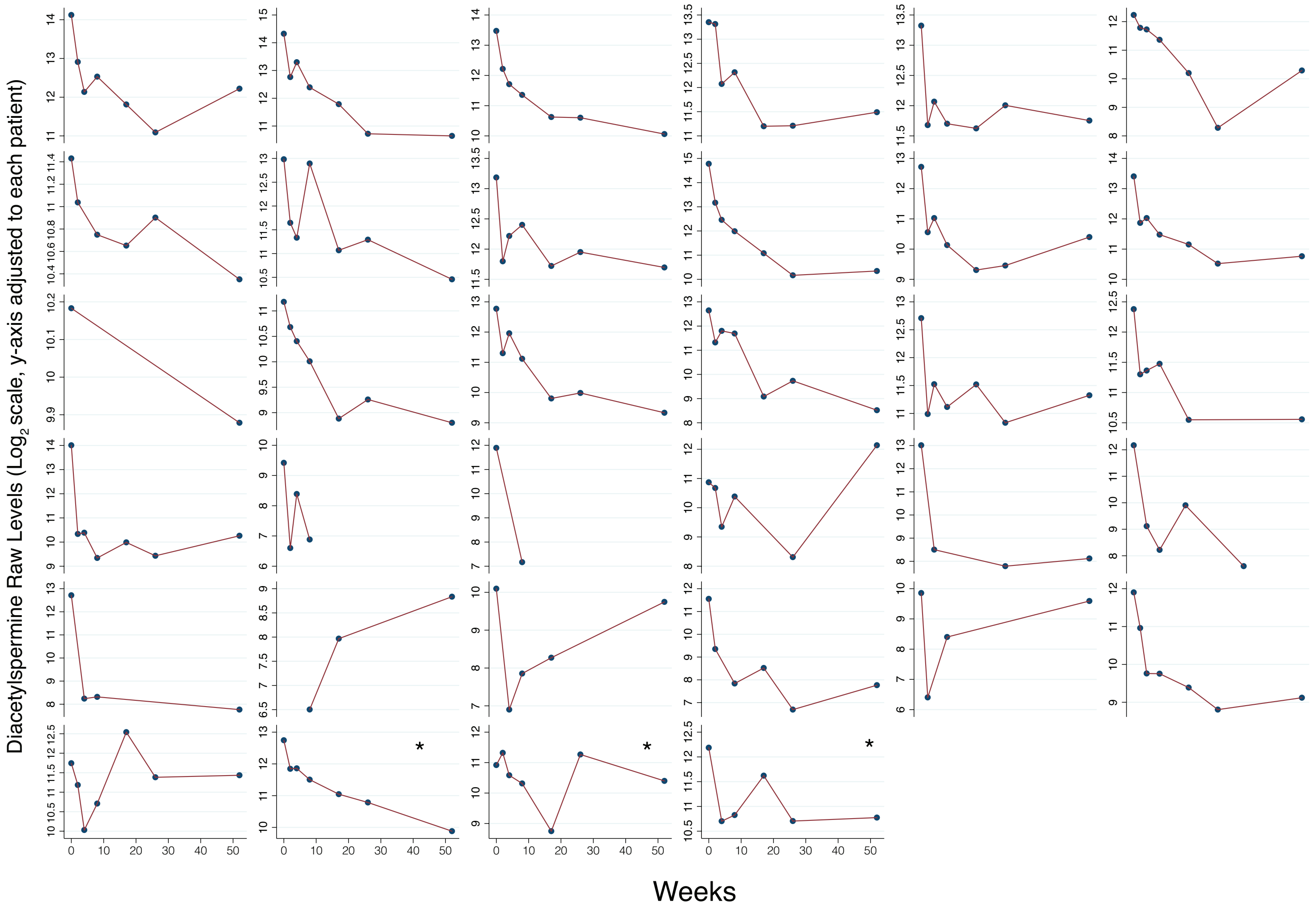

Figure S5. Raw Urinary Abundance of DiAcSpm by Patient in CTB2 Cohort (n=34). Graphs show DiAcSpm trend profiles over time of each patient from the CTB2 cohort. Trends are generated using raw data from mass spectrometry analysis, all of which have been normalized to respective urinary creatinine values. Y-axes have been adjusted to mass spectrometry abundance ranges of each patient due to machine variations from different runs. Missing values in select graphs represent missing samples or samples that were already below 150mOsMs prior to dilution. The last three graphs marked with "*" represents graphs of patients who showed repeat positive culture or AFB scores at week 52 after treatment termination (Table S1). Lack of clinical data informing whether the positivity at 52 weeks was due to re-infection, incomplete treatment, or other causes prevents meaningful interpretation of these findings at this time.

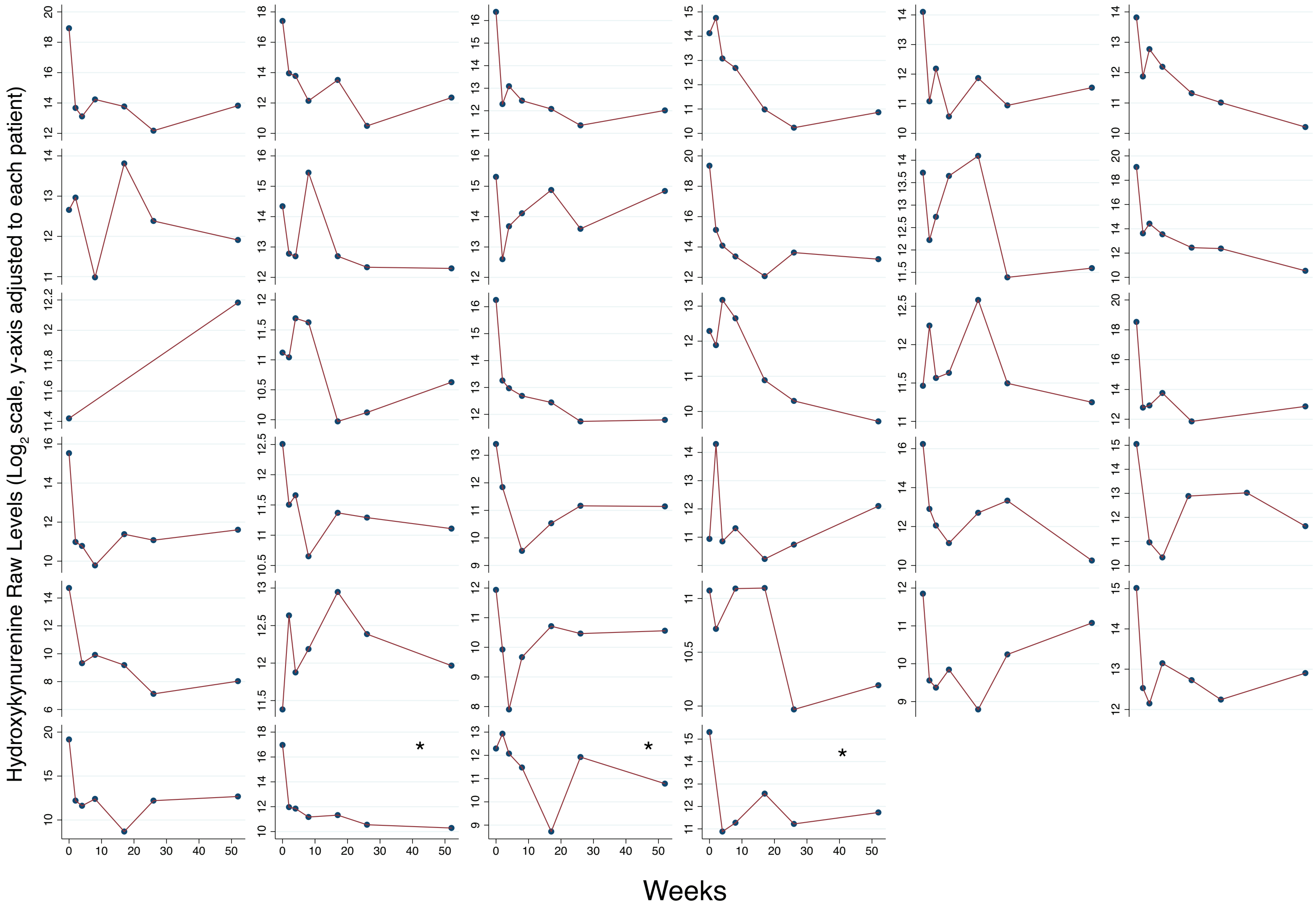

Figure S6. Raw Urinary Abundance of Hydroxykynurenine by Patient in CTB2 Cohort (n=34). Graphs show Hydroxykynurenine trend profiles over time of each patient from the CTB2 cohort. Trends are generated using raw data from mass spectrometry analysis, all of which have been normalized to respective urinary creatinine values. Y-axes have been adjusted to mass spectrometry abundance ranges of each patient due to machine variations from different runs. Missing values in select graphs represent missing samples or samples that were already below 150mOsMs prior to dilution. The last three graphs marked with "*" represents graphs of patients who showed repeat positive culture or AFB scores at week 52 after treatment termination (Table S1). Lack of clinical data informing whether the positivity at 52 weeks was due to re-infection, incomplete treatment, or other causes prevents meaningful interpretation of these findings at this time.

Table S1. Sputum AFB and culture status of CTB2 participants (n=34)

Week 0		Week 4		Week 8		Week 26		Week 52	
Culture	AFB	Culture	AFB	Culture	AFB	Culture	AFB	Culture	AFB
+	3+	+	2+	+	0	-	0	-	0
+	2+	+	2+	+	0	-	0	-	0
+	4+	+	3+	+	3+	-	0	-	0
+	4+	+	3+	-	2+	-	0	-	0
+	3+	-	2+	-	0	-	0	-	0
-	4+	+	0	-	0	-	0	+	0
+	3+	+	Scanty	-	0	-	0	-	0
+	2+	+	0	+	Scanty	-	0	-	0
+	Scanty	-	0	-	0	-	0	-	0
+	1+	+	1+	-	Scanty	-	0	-	0
-*	0	-	0	Contam	0	-	0	-	0
+	3+	+	1+	+	1+	-	0	-	0
+	1+	+	0	-	0	-	0	-	0
+	Scanty	-	0	-	0	-	0	-	0
+	1+	+	1+	-	0	-	0	-	0
+	Scanty	-	0	-	0	-	0	-	0
+	3+	+	Scanty	-	Scanty	-	0	-	0
+	3+	+	0	+	Scanty	-	0	-	0
+	3+	+	2+	+	0	-	0	+	Scanty
+	3+	-	0	-	0	-	0	+	0
Contam	2+	+	2+	Contam	0	-	0	-	0
+	1+	Contam	Scanty	-	0	-	0	-	0
+	1+	+	Scanty	-	Scanty	-	0	-	0
+	1+	+	Scanty	-	Scanty	Contam	0	-	0
+	Scanty	+	Scanty	+	0	-	No data	-	0
+	0	-	0	-	0	-	0	-	0
+	Scanty	+	Scanty	+	0	-	0	-	0
+	2+	-	0	+	0	-	0	-	0
+	1+	+	0	Contam	0	-	0	-	0
-*	0	-	2+	-	0	Contam	0	-	0
+	0	+	0	-	0	-	0	-	0
+	0	+	0	-	0	-	0	-	0
+	Scanty	-	0	Contam	0	-	0	-	0
+	Scanty	+	Scanty	Contam	0	-	0	-	0

Each row represents data from a single patient

* Diagnosis based on positive GeneXpert at Week 0
